# Controlling photosynthetic energy conversion by small conformational changes

**DOI:** 10.1111/ppl.13802

**Published:** 2022-11-03

**Authors:** Naama Maroudas‐Sklare, Yuval Kolodny, Shira Yochelis, Nir Keren, Yossi Paltiel

**Affiliations:** ^1^ Department of Applied Physics Hebrew University of Jerusalem Jerusalem Israel; ^2^ Department of Plant & Environmental Sciences, The Alexander Silberman Institute of Life Sciences Hebrew University of Jerusalem Jerusalem Israel

## Abstract

Control phenomena in biology usually refer to changes in gene expression and protein translation and modification. In this paper, another mode of regulation is highlighted; we propose that photosynthetic organisms can harness the interplay between localization and delocalization of energy transfer by utilizing small conformational changes in the structure of light‐harvesting complexes. We examine the mechanism of energy transfer in photosynthetic pigment‐protein complexes, first through the scope of theoretical work and then by in vitro studies of these complexes. Next, the biological relevance to evolutionary fitness of this localization‐delocalization switch is explored by in vivo experiments on desert crust and marine cyanobacteria, which are both exposed to rapidly changing environmental conditions. These examples demonstrate the flexibility and low energy cost of this mechanism, making it a competitive survival strategy.

## 1 INTRODUCTION

The biological realm is rarely associated with truly quantum or coherent effects, as these are deemed too sensitive to manifest in the relatively high temperatures and larger scales associated with biology. There is, however, growing evidence that certain quantum effects play a role in some biological processes. One example is the radical pair mechanism (RPM) suggested to be involved in magnetic field sensing for bird navigation; another is the exciton energy transfer (EET) in photosynthetic light‐harvesting systems (Lambert et al., 2012; Rodgers & Hore, 2009). Such examples are still controversial, for instance, alternative hypotheses to the RPM have been proposed for bird navigation (Natan & Vortman, 2017; Werber et al., 2022).

Photosynthesis is the backbone of life as we know it; it is the engine converting solar energy into chemical energy stored in sugars that organisms use to grow and multiply. Absorbed solar energy is a double‐edged sword: without it, photosynthesis is nonexistent and life grinds to a halt, but on the other hand, an excess of energy can be highly destructive (Taiz et al., 2015). Unregulated absorption of solar energy can give rise to harmful oxidative radicals, which damage membranes and proteins or even the DNA of an organism. As such, the regulation of solar absorption and dissipation is key in maintaining homeostasis in photosynthetic (Cruz et al., 2004; Taiz et al., 2015). In certain conditions, the quantum yield of photosynthesis can be extremely high, up to 0.8 for the initial reaction (Barber, 2007; Nelson & Yocum, 2006). The quantum yield is a measure of efficiency, the number of photons successfully converted to chemical energy divided by the number of photons absorbed. Even so, the efficiencies measured in natural environments are normally much lower (Barber, 2007). This can be puzzling at first glance. However, it is reasonable to assume that efficiency is sacrificed in favor of increased stability and robustness. The mechanisms dictating energy transfer efficiencies are under intense study but are far from being fully understood, classical models, for instance, predict even lower efficiencies than experimentally observed values (Chenu et al., 2017). Light harvesting complexes, though ranging in size and composition, exist in the realm where both quantum and classical energy transfer is feasible. This review explores the possibility that rapid switching between different organization regimes is employed as a means of energy transfer regulation. The switching between coupling domains provides control over light harvesting efficiency that can be achieved just by small conformational changes.

Several experimental results support the existence of coherent effects in light harvesting complexes (coherent: Box 1‐ Baumgratz et al., 2014; Young, 1804) (experimental results: Engel et al., 2007; Herek et al., 2002; Hildner et al., 2013; Rathbone et al., 2020; Romero et al., 2017; Schlau‐Cohen et al., 2013, 2015), but there is still an ongoing debate over whether they are indeed fully quantum or not (Keren & Paltiel, 2018). For example, although 2D electron spectroscopy of a photosynthetic pigment‐protein complex exhibited picosecond time scale beats (Engel et al., 2007), these can be interpreted in two ways. Either they represent electronic coherence from the quantum properties of the wave packet, or they are a result of the coupling of the excitation to mechanical vibrations of the pigment in the protein matrix (Cao et al., 2020; Keren & Paltiel, 2018; O'Reilly & Olaya‐Castro, 2014; Runeson et al., 2022; Thyrhaug et al., 2018). These discussions often boil down to a question of scale—what frequency would be relevant to a quantum effect versus a classical one? This review does not attempt to distinguish between the two, rather, it aims to show how changes in coupling and, therefore, coherence can be utilized to shift between quenching and harvesting of solar energy. This is similar to coupling control, as seen in waveguides and light propagation, where enhanced coupling leads to delocalization of the wave packet (Gilead & Silberberg, 2017). The inherent disorder of the system and the intermediate coupling between pigments makes it possible to swap between localized and delocalized energy states.

BOX 1 **What do we mean by “coherent”?**

In physics, coherence describes a constant difference between the phases (angles) of waves that have the same frequency. Coherent sources can create interference patterns, while incoherent sources, which have a changing relation between their phases, cannot. The easiest way to create two coherent sources is to split a single source of coherent light, similarly to the famous double‐slit experiment which showed the dual nature of light.

**Coherence can refer to a completely classical phenomenon of wave interference, but it can also be used in a quantum sense to differentiate between “pure” and “mixed” states.** The laser, superconductivity and superfluidity are all macroscopic phenomena that arise from a highly coherent state in certain quantum systems. Coherent control of quantum systems has recently been formulated as a resource theory by Romero et al. (2017) showing that the relevant resources of entanglement and coherence are found to be equivalent and closely related to a measure of discord.

**FIGURE 1:**
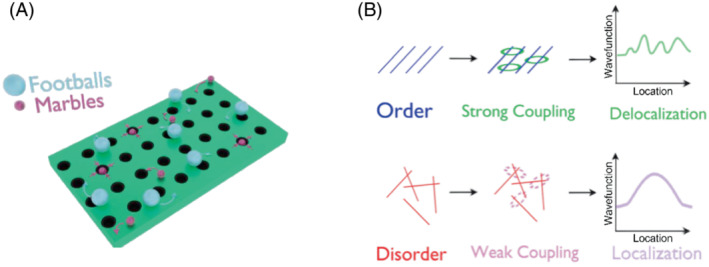
(A) Graphical representation of the “football field” explanation for localized versus delocalized states. The marbles fall into the holes and are localized, while the footballs pass over them and are thus delocalized over the whole field. (B) In many of the systems studied, order generates stronger coupling between subunits, leading to delocalization effects. On the other hand, disorder leads to weaker coupling and localization in the system. The x axes in each of the graphs represent the 1D reduction of 3D space. The y axes represent the wavefunction of the exciton, i.e., the probability density in location space. Thus, delocalization is represented by a disseminated wavefunction in space, while localization is represented by a noticeably higher probability density in a specific location.

Many different definitions are used for the terms “coherent” or “delocalized”; we will adhere to the definition of Chenu and Scholes (2015) who define coherent EET as the regime between the Förster and Redfield limits (Novoderezhkin et al., 2004) (Box 2; Schneckenburger, 2020; Seibt & Mančal, 2017). This is relevant because photosynthetic light harvesting occurs mostly in this regime (Schneckenburger, 2020). To use an excellent explanation attributed to Prof. Rienk van Grondelle (Figure 1A), we can imagine the energetic landscape of the photosystem as a football field with holes the size of ping pong balls. Localized states will be the size of marbles and easily get stuck in one of the holes. Footballs, on the other hand, will pass over the holes and move freely around the field—these will be delocalized states. Furthermore, delocalized states will not be associated with phase coherence, rather, they will define an electronic excited state comprised of a superposition of excitations at different molecular sites. Another useful definition for this article is the photosynthetic unit (Mauzerall & Greenbaum, 1989), defined as the sum total of pigments that contribute excitation to a single reaction center. A reaction center (RC) is the location of the photochemical charge separation reaction in the photosystem, which is the culmination of EET between the pigments, and the driving force behind the conversion of light energy to chemical energy.

BOX 2 **Two regimes of energy transfer: FRET vs Redfield**

Förster resonance energy transfer (FRET) is based on the theory of non‐radiative energy transfer presented by Theodor Förster in 1948. It describes resonant energy transfer by dipole–dipole interaction from a donor to an acceptor molecule. Due to the R^−6^ dependence on molecular distances for dipole–dipole interactions, **FRET is considered relevant in distances of around 5 nm**.

Redfield theory is derived from the Redfield Equation, which describes the decay of excitations in a quantum system due to external fluctuations, in a model with weak system‐bath interaction. **The relevant distances for Redfield energy transfer are sub‐nanometer**, as befit a quantum theory. Since Redfield transfer is influenced by energy gaps, for small enough gaps the Redfield rates are higher than the Förster rates.

To date, numerous examples have exhibited delocalization effects in photosynthesis. Such examples provide us with the scale over which we can expect to see such effects. In 2007, Engel et al. (2007) showed quantum beats in the 2D spectra of the Fenna–Matthews–Olson (FMO) protein‐bacteriochlorophyll complex. This complex is found in green sulfur bacteria and consists of three subunits with eight bacteriochlorophylls (BChl, a type of pigment) each (Ben‐Shem et al., 2004; Camara‐Artigas et al., 2003; Fenna & Matthews, 1975; Li et al., 1997). Further research using 2D echo spectroscopy has provided evidence for quantum coherent sharing of electronic excitation in isolated antenna complexes from marine cryptophytes (Collini et al., 2010). A subsequent study demonstrated the relation between protein‐pigment complex structure and the coherent coupling between pigments in that structure by comparing the “open” and “closed” configuration of phycobiliprotein antennae in related cryptophyte species—*Rhodomonas*, *Chroomonas*, and *Hemiselmis* (Harrop et al., 2014). Another important finding is the ability of single light harvesting (LH) complexes to “blink” between energy‐transferring and energy‐quenching states (Chmeliov et al., 2013; Schlau‐Cohen et al., 2013, 2015). Two types of LH complexes were found to exhibit this behavior. These are the LH2 complex, which has a ring‐like structure composed of nine subunits with three BChl and one carotenoid each, and the LHCII complex, which contains three subunits with 14 chlorophyll and four carotenoids each. Additionally, long‐lived coherences of at least 400 femtoseconds were observed by Hildner et al., 2013 on single LH2 complexes, following previous work on quantum coherent control in this system (Herek et al., 2002). All examples given are remarkable but refer to complexes much smaller than a whole photosynthetic unit. We can therefore assume that the energy transfer of a whole photosynthetic unit will exhibit more complex behavior (Romero et al., 2017).

An important point to address is the dimensionality of the energy transfer path. Anderson localization dictates that in 1D, localization will inevitably occur due to the weak interaction between neighboring points in a lattice (Anderson, 1958) (Box 3). If we assume that energy transfer takes place along a given 1D path, this will prescribe localization and quenching. So, to achieve large enough delocalization, one can either enhance coupling or add another degree of freedom to the energy transfer path.

BOX 3 **What is Anderson localization?**

In 1958, P.W. Anderson published a paper showing that **even a small amount of disorder can lead to localization in a system with low dimensionality**. The paper presented a simple model for processes such as spin diffusion or impurity band conduction. In these processes, transport occurs by quantum‐mechanical “jumps” between neighboring points in a random lattice. It was shown that at sufficiently low densities, transport does not take place at all, and the exact wave functions are localized in a small region of space.

In the model, 𝑉 is the interaction matrix element between neighboring sites in a 3D lattice. **The higher the disorder in a system, the weaker the interaction between the sites**. If *V* falls off faster than 1/𝑟^3^ and the average value is smaller than a certain critical value 𝑉_0_, then there can be no diffusion. Therefore, when applying the theory to 1D, it can be shown that localization must occur, no matter how small the disorder is.

Though the mechanism of energy transfer is a fascinating subject in and of itself, from a biological perspective, an even more relevant issue should be addressed. As put forth by Chenu and Scholes (2015), the main question in this discussion is whether long‐lived delocalization in photosystems confers an evolutionary advantage to these organisms. It is possible that the biological world has harnessed a toolkit of rapid swapping between localization and delocalization that increases fitness. There are many different structures of pigment‐protein complexes in nature, and even within the same organism, these structures can change depending on external stimuli (A.W.D. Larkum, 2003; Rathbone et al., 2020; Torres et al., 2014). The importance of this diversity may be in creating the possibility for operating at specific energy transfer regimes that will best suit a given set of environmental conditions. This paper will attempt to answer this central question by reviewing data from organisms, cyanobacteria, in particular, subjected to fast‐changing conditions, who appear to utilize the localized–delocalized switch to adapt to these changes swiftly and efficiently.

## 2 DELOCALIZATION: FROM THEORY TO PRACTICE

**FIGURE 2:**
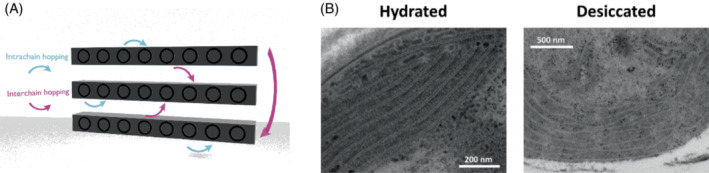
(A) In the theoretical model presented by Mendoza‐Arenas et al., hopping of excitons inside the same chain (intrachain) and between chains (interchain) is allowed. In addition, coupling between the first and third chains creates an infinite loop model. Enhanced interchain coupling enables highly efficient exciton transfer. The physical explanation behind enhanced coupling leading to greater conductivity is that the energy of the bound states is broken down into states of lower potential energy, but much higher kinetic energy, so that the excitons can travel longer distances. (B) TEM images of desert cyanobacteria. Note the ordered structure in hydrated samples versus the disordered, aggregated state in the desiccated sample. The above images were produced by the late Prof. Itzhak Ohad in the Weizmann institute electron microscopy unit. We would like to thank Dr. Reinat Nevo for sharing them with us.

An integral part of our understanding of how these mechanisms might work comes from theoretical work by Mendoza‐Arenas et al. (2014). Their model consists of three incoherently coupled 1D quantum spin chains, where the first and last chain interact as well, creating a loop analogous to infinitely coupled chains. Both intrachain (within the same chain) and interchain (between adjacent chains) hopping of excitons is allowed (Figure 2A). The study showed that with sufficient interchain hopping, overall transport of excitons is greatly enhanced. Furthermore, this effect increases with the length of the chains, demonstrating the relevance of this mechanism for bulk materials. Levi et al. (2011) found that a certain amount of disorder in quasi‐crystals enhances transport, while too much disorder results in localization. Interpreting the disorder in this experimental result in terms of increased interchain coupling supports the theoretical model and provides a physical example of what is thought to occur in biological systems. The central point that needs to be considered when applying this theory to photosynthesis is that one small change is enough to control an entire system. A minuscule area can be either coupled or uncoupled to its neighbor, leading to either delocalization and exciton transfer or localization and energy dissipation. Thus, the physical configuration of the antenna pigments themselves becomes the key point of control for the entire photosynthetic unit.

The next step in understanding localization and delocalization effects is elucidated by in vitro systems fabricated and studied by Eisenberg et al. (2014). They observed that units of a single type of pigment‐protein complex, phycocyanin (PC), when isolated and dried, spontaneously create ordered structures of nanowires that can be hundreds of microns long. The exciton lifetime is shortened by a factor of three in these dried, organized structures compared with the same pigments in solution, which could indicate more efficient energy transfer. The theory explained earlier fits here nicely—the more structured the system, the longer the rods are expected to be, in agreement with the theoretical calculation of a positive correlation between length and energy transfer efficiency. In addition, near‐field scanning optical microscopy (NSOM) measurements indicated long range excitonic transfer and strong transverse (interchain) coupling in ordered structures. These results link to the theoretical explanation of delocalization by increased interchain coupling.

Moving on to in vivo systems, we can gain insights by looking at work done by Bar‐Eyal et al. (2015, 2017) on desert crust cyanobacteria (Figure 2B). The photosynthetic organisms studied are subject to conditions of extreme desiccation in their natural environment on the surface of sand dunes. During the night, there is limited condensation of dew on the surface, and cyanobacteria must make the most of the short time window in the morning when conditions are amenable to photosynthesis—there is enough sunlight for photosynthesis, and the moisture has not dried up yet. When desiccation does happen, they quickly change phase and block photosynthesis, while thermally dissipating very high amounts of incident light energy. The transitions between desiccated and hydrated states happen incredibly fast—much faster than the time scales needed to change gene expression and generate specialized proteins to deal with the new conditions. In the experiments, desiccated samples of the cyanobacterium, in comparison with hydrated samples, exhibited a significant decrease in energy transfer, as determined by the fluorescence lifetimes versus spectra maps obtained by Streak camera experiments. The explanation for this is that desiccation results in increased aggregation and disorder of the pigment rods in the photosynthetic antennae (Figure 2B), which enhances energy transfer along the rods and in between them, without reaching the reaction center. Unintentional excitation of the RC in a desiccated cyanobacterium can be extremely harmful: since there is no terminal acceptor for the energy in this state, excitation could result in harmful free radicals. In this case, increased disorder leads to more efficient heat dissipation over long energy transfer paths. Upon rehydration, the antennae revert to their more ordered state within seconds and resume their ability to transfer exciton energy to the RC. So, we see an intuitive example where structural changes allow quick switching between localized and delocalized energy transfer, efficiently protecting the organisms and allowing them to cope with a challenging environment.

Another intriguing example of this mechanism in vivo was shown by Kolodny et al. (2020) in their research on marine cyanobacteria. Marine *Synechococcus* must adapt to quickly changing light conditions in their natural environment. They are unable to control their position in a mixing water column and can therefore find themselves rapidly transported from deep, dark waters to the bright surface, or vice versa. The studies found that cyanobacteria grown in low‐light (LL) conditions had longer pigment rod structures than those grown in high‐light (HL) conditions. In conjunction with longer rods, shorter fluorescence lifetimes were observed in the LL samples. Theoretical work by Chenu et al. (2017) calculated that the optimal PC rod is 2–4 trimers long, due to the trade‐off between increased rod length for enhanced light harvesting and decreased length for efficient EET (assuming 1D energy transfer). Taking this into account, the shorter lifetimes in longer rods mean that either delocalization occurs over much larger distances than previously observed or, more plausibly, that EET occurs in more than one dimension. The latter explanation ties in neatly with the theoretical work of Mendoza‐Arenas et al. (2014), and provides another in vivo example of where this mechanism can function in nature. Moreover, when cyanobacteria are rapidly transported from LL to HL regimes, they can quickly disconnect their large antennae from the RC and, in doing so, protect the RCs from excessive EET, which, as mentioned before, can result in harmful free radicals. Again, we see quick exchanges from delocalization to localization that protect the organisms and increase their survivability. Structural models based on cryo‐transmission electron microscopy (TEM) images of the photosynthetic antenna in another species of cyanobacteria and in red algae present dense LH antennas connected by interchain molecular linkers (M. Li et al., 2021; Rast et al., 2019), in such a way that would enable this kind of flexible interplay between energy dissipation and utilization.

Finally, we arrive at in vivo research done in the field: recent research conducted by Kolodny et al. (soon to be published in Nature Comm. Bio.) shows that the phenomena of decreased fluorescence lifetime in LL marine photosynthetic organisms, hold in a natural setting as well. Stratified waters from the Gulf of Aqaba at Eilat were sampled at intermittent depths, and measurements revealed that the fluorescence lifetime decreased as depth increased (which corresponds to decreasing levels of light). Stepping out of the lab and into the field greatly increases the relevance of these findings by demonstrating the applicability of the theory proposed here to a much wider range of photosynthetic organisms in their native environment.

## 3 CONCLUSION

**FIGURE 3:**
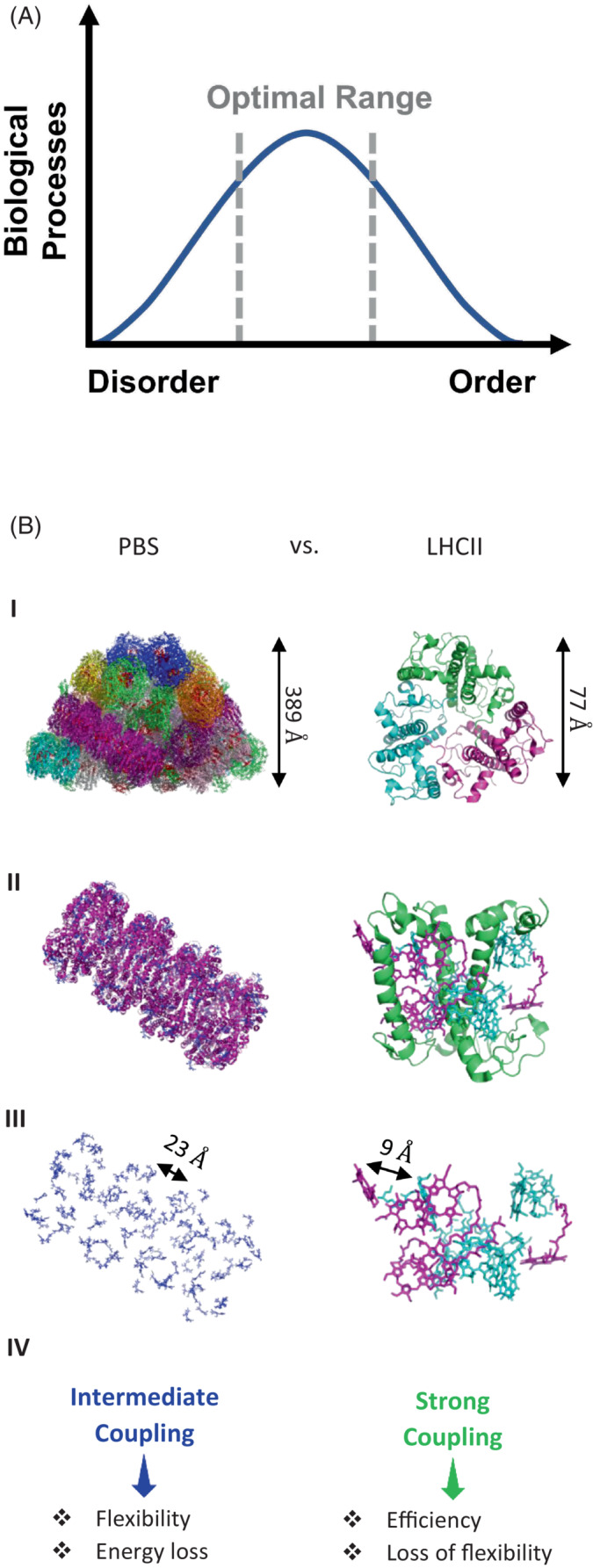
(A) In biology, both efficiency (associated with order) and flexibility (associated with disorder) are important for survival. Thus, the optimal range for biological processes lies in between maximum disorder and maximum order. (B) Two examples of LH structures in photosynthetic organisms, PBS and LHCII. Presented are the whole antenna structure (I), magnification of a single subunit (II), and the pigments alone (III). Each LH structure exhibits different mechanisms that are optimal for each organism's environment (IV). Phycobilisome (PBS) is a LH complex found in cyanobacteria and red algae, which need flexibility to adapt to unpredictable and rapid changes in conditions. Light harvesting complex II (LHCII) is a LH complex found in land plants and green algae. It exhibits more efficient energy transfer but lacks flexibility. These two structures represent two different options in the range of energetic coupling observed in photosynthetic organisms—note the differences in typical distances between the two structures. Our thanks to Emma‐Joy Dodson for creating these structural images.

In this review, we examined delocalization in biological systems fulfilling a new role—control of light harvesting efficiency. Interaction between small coherent domains enables flexibility and quick conversion between energy transfer and dissipation. In analogy to allosteric changes in a protein, small changes enable loss or regeneration of delocalization in larger domains, quickly and reversibly channeling energy transfer in the system. Minute changes at low energy cost to the organism can result in large effects, making the process both efficient, reversible, and flexible (Figure 3A). Moreover, both lab and field examples show that this mechanism can indeed enhance adaptation and confer an evolutionary advantage to photosynthetic organisms exposed to extreme changes in environmental conditions.

As mentioned, there is great variability in the structural composition and arrangement of photosystems in nature (Figure 3B) (Qian et al., 2021). For instance, the antennae of *Acharyochloris marina* are composed of long, straight rods of PC, which assist in preferential harvesting of far‐red light (Rast et al., 2019). Purple bacteria, for their part, use chromatophores, which are pseudo‐organelles that contain pigment‐protein complexes for light harvesting and RCs (Chandler et al., 2014). Land plants have thylakoid membranes which integrate specific protein‐pigment complexes at significantly different locations, allowing for step‐wise control of the photosynthetic process (Johnson et al., 2014; Walker et al., 2020). All the above can potentially use the switch from localized to delocalized EET in a myriad of interesting and yet unknown ways. In contrast, green sulfur bacteria that live in the deep ocean and are exposed to minimal amounts of light, have crystalline‐like structures of pigments called chlorosomes, which are inherently inflexible (Pšenčík et al., 2009). However, since the changes in conditions and light intensity are minimal and far less drastic than for the other organisms discussed here, this would not be a major limitation. This highlights the difference between extreme static conditions and extreme dynamic conditions. An intriguing new front to explore in this sense would be photosynthetic organisms exposed to extreme temperature changes, where flexibility and rapid adaptation would be key components in an organism's success and proliferation.

## AUTHOR CONTRIBUTIONS

NK and YP conceived the initial idea, NMS wrote the manuscript and interpreted the relevant literature, NMS, SY, NK and YP designed the outline and conceptualized the figures. All authors revised the manuscript and provided critical feedback for content and style. We thank Hammam Al‐Bustami for graphical work.

## Data Availability

Data sharing is not applicable to this article as no new data were created or analyzed in this study.
